# Overexpression of platelet-derived growth factor receptor alpha promotes tumor progression and indicates poor prognosis in hepatocellular carcinoma

**DOI:** 10.18632/oncotarget.2537

**Published:** 2014-10-17

**Authors:** Tao Wei, Li-Na Zhang, Yi Lv, Xiao-Ya Ma, Lei Zhi, Chang Liu, Feng Ma, Xu-Feng Zhang

**Affiliations:** ^1^ Department of Hepatobiliary Surgery and Institute of Advanced Surgical Technology and Engineering, the 1st Affiliated Hospital of Medical College, Xi'an Jiaotong University, Xi'an, Shaanxi Province, 710061, China; ^2^ Department of Pharmacy, the 2nd Affiliated Hospital of Medical College, Xi'an Jiaotong University, Xi'an, Shaanxi Province, 710004, China; ^3^ Department of General Surgery, General Hospital of Ningxia Medical College, Yinchuan, Ningxia Hui Autonomous Region, 750004, China; ^4^ Department of Hepatobiliary and Pancreatic Surgery, The Second Affiliated Hospital, Zhejiang University School of Medicine, Hangzhou, 310009, China

**Keywords:** Platelet-derived growth factor receptor alpha, Hepatocellular carcinoma, survival, metastasis, CD31

## Abstract

Dysregulation of platelet-derived growth factor receptor alpha (PDGFRα) has been documented in various cancers. However, its role in hepatocellular carcinoma (HCC) remains unknown. We and others have examined that upregulation of PDGFRα might be involved in hepatocarcinogenesis. Here, we report that PDGFRα plays a critical role in HCC progression and prognosis. The expression of PDGFRα was markedly higher in human HCC compared to adjacent liver tissues. Although PDGFRA mRNA was decreased in HCC, PDGF-A mRNA was dramatically increased in HCC. Overexpression of PDGFRα was strongly correlated with microvessel density (MVD) of HCC (p<0.05), as well as macroscopic vascular invasion of the tumors (p<0.05). HCC patients with high PDGFRα expression displayed a shorter overall survival and a higher recurrence rate than those with low PDGFRα expression (p<0.05, respectively). Additionally, stable overexpression of PDGFRα in hepatoma cells promoted cell proliferation, migration, invasion and epithelial-mesenchymal transition in vitro. Similarly, an in vivo assay showed that PDGFRα overexpression in hepatoma cells exhibited remarkably tumorigenic potential in tumor size and weight in vivo, which displayed markedly elevated MVD than controls. Thus, our study provided the evidence that PDGFRα may serve as a candidate prognostic marker and a novel therapeutic target for HCC.

## INTRODUCTION

Molecular therapies have gained wide acceptance in tackling malignancies along with accumulated understanding of molecular and cellular mechanisms regulating tumor growth and progression. In particular, interplay between growth factors and cognate receptors is recognized as a major contributor in carcinogenesis, and blocking theses interactions have been introduced with marked efficacy in preclinical models and clinical settings [1, 2]. Hepatocellular carcinoma (HCC) is commonly known to be notoriously resistant to systematic chemotherapies. But the advent of sorafinib, which is an oral multiple receptor tyrosine kinase inhibitor and exhibits remarkable survival benefit for advanced unresectable HCC [3, 4], brings hope that therapeutic agents could be efficacious in this intractable condition and encourages further development of novel targeted therapeutic approaches.

Platelet-derived growth factor (PDGF) family represents as a prototype of growth factor function. Specifically, four PDGF ligands, namely PDGF-A, -B, -C and -D, differentially bind with two distinct receptor isoforms, PDGF receptor α (PDGFRα) and -β, which subsequently activate their downstream signaling cascade and induce various cellular responses [5, 6]. The molecular pathogenesis mediated via PDGFRα has been sparsely documented in liver development and hepatocarcinogenesis [7-12]. Our previous work revealed that β-catenin (a well-known oncogene) knockout mice had an unexpected increase of HCC development and the main contributing factor for this paradox was an escaping signaling pathway through PDGFRα/PI3K/Akt [13]. The *in vitro* studies corroborated that expression of PDGFRα remarkably increased when β-catenin was inhibited, indicating that PDGFRα and β-catenin might account for two divergent carcinogenic mechanisms and distinct subgroups of HCC patients [13]. In the current study, we present the evidence that PDGFRα expression promotes HCC growth, invasion and metastasis. Additionally, we show that PDGFRα overexpression predicts poor prognosis in HCC patients undergoing curative resection.

## RESULTS

### PDGFRα is upregulated in human HCC tissues

**Figure 1:**
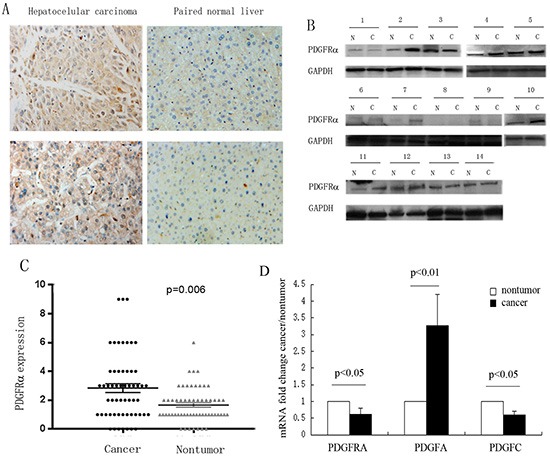
Increased expression of PDGFRα was detected in hepatocellular carcinoma (HCC) **(A)** Representative IHC staining showed strong PDGFRα expression in human HCC than the adjacent normal liver tissues. Original magnification: 40×. **(B)** Protein was extracted from the 14 matched HCC and adjacent liver tissues and subjected to western blot analysis to examine different expression levels of PDGFRα. **(C)** Expression level of PDGFRα was scored and compared between HCC and adjacent liver tissues. **(D)** Comparison of mRNA expression of PDGFRα, PDGF-A and PDGF-C between HCC tumor and non-tumor tissues.

To explore clinicopathological significance of PDGFRα expression in HCC, we firstly investigated PDGFRα protein expression in 57 pairs of human HCC and adjacent normal liver tissues by immunohistochemistry (IHC) analysis. IHC assays showed that PDGFRα expression was mainly localized to cell membrane and cytoplasm. High protein level of PDGFRα was seen in 22 out of 57 (38.6%) HCC tissues, compared to only 4 out of 57 (7.0%) adjacent liver tissues (*p*=0.006) (Figure 1 A and C). Additionally, upregulation of PDGFRα protein was confirmed in 14 paired human HCC specimens by western blot analysis (Figure 1B). 5 out of 14 (35.7%) HCC displayed high PDGFRα protein level compared to the adjacent normal tissues. Intriguingly, the mRNA level of PDGFRA was lower in HCC versus normal liver (*p*<0.05). To investigate the potential mechanism of PDGFRα upregulation in HCC, we further detected the expression of its ligands, PDGF-A and -C. It was found that PDGF-A but not C chain was dramatically upregulated in tumors compared to normal liver (p<0.01). These data indicates that PDGFRαis activated in HCC probably in an autocrine or paracrine fasion.

### Correlation of PDGFRα expression and clinicopathological characteristics of HCC

To further investigate whether high expression of PDGFRα is related to HCC progression, the characteristics of the 57 HCC patients were analyzed. The expression of PDGFRα was assessed by IHC and defined scoring system. The correlation of PDGFRα expression and clinicopathological factors are shown in Table 1. Although no significant correlation was observed between PDGFRα expression and liver cirrhosis, tumor size, number, differentiation or alpha-fetoprotein level (*p*>0.05), statistically significant correlation between PDGFRα expression and macroscopic vascular invasion (MVI) were found (*p*=0.010). Spearman analysis also revealed that high PDGFRα expression was positively correlated with vascular invasion (r=0.212, *p*<0.001).

**Table 1:** Correlation of PDGFRα expression with clinicopathological characteristics of 57 hepatocellular carcinoma specimens

variables	NO.	PDGFRα expression		*p* value
		High (%)	Low (%)	
Cirrhosis Present Absent	4611	20 (43.5) 2 (18.2)	26 (56.5) 9 (81.8)	0.122
Tumor size <5cm ≥5cm	17 40	6 (35.3) 16 (40.0)	11 (64.7) 24 (60.0)	0.738
Tumor number Solitary Multiple	48 9	18 (37.5) 4 (44.4)	30 (62.5) 5 (55.6)	0.695
Vascular invasion Present Absent	13 44	9 (69.2) 13 (29.5)	4 (30.8) 31 (70.5)	**0.010**
Histology differentiation Poorly Moderately Well	3 37 17	0 (0) 14 (37.8) 8 (47.1)	3 (100) 23 (62.2) 9 (52.9)	0.300
Alpha-fetoprotein <200 ng/ml ≥200 ng/ml	12 45	2 (16.7) 20 (44.4)	10 (83.3) 25 (55.6)	0.079

### High PDGFRα expression predicts poor prognosis of HCC after curative surgery

**Figure 2:**
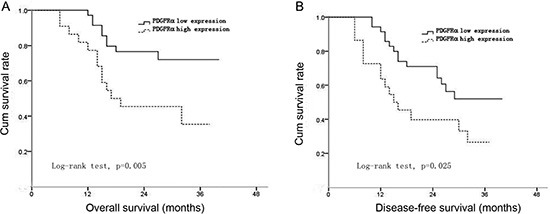
The correlation between PDGFRα expression and overall survival (OS) or disease-free survival (DFS) of the patients after surgery Curves of OS **(A)** and DFS **(B)** were plotted by Kaplan-Meier methods according to PDGFRα expression levels.

The survival curves after surgery of HCC patients according to PDGFRα expression were plotted by Kaplan-Meier methods. The results showed patients with high PDGFRα expression had significantly worse overall survival (OS) and disease-free survival (DFS) than those with low PDGFRα expression (log-rank test, *p*=0.005, and *p*=0.025, respectively) (Figure 2A and B). These findings indicated that HCC patients with high PDGFRα expression had higher risk of tumor recurrence and shorter survival time even after curative surgery.

### Analysis of PDGFRα expression in vascular invasion of HCC

**Figure 3:**
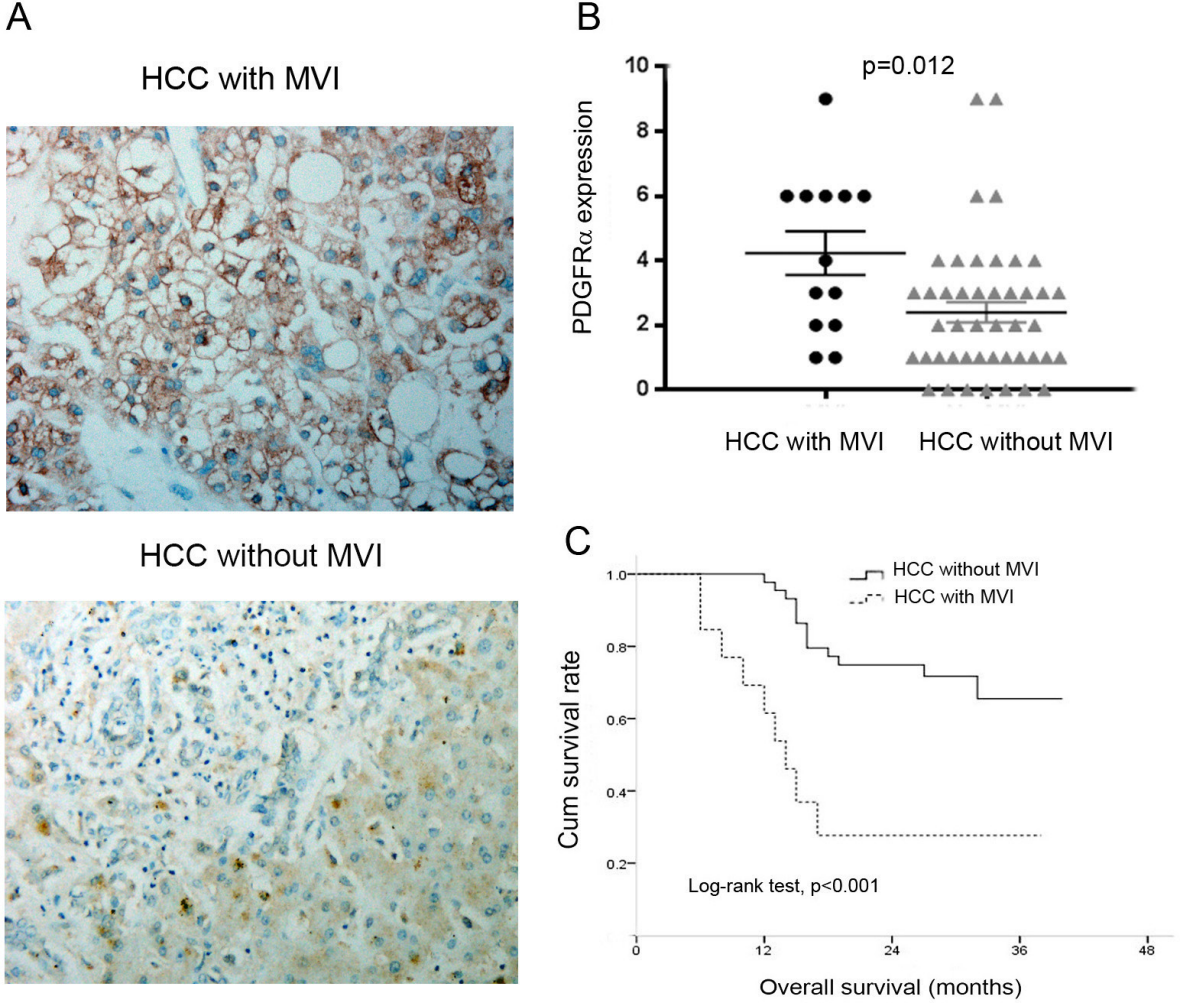
The expression of PDGFRα was analyzed according to macroscopic vascular invasion (MVI) status **(A)** Representative IHC staining showed strong PDGFRα expression in primary HCC with MVI than that without MVI. Original magnification: 40×. **(B)** Quantitative analysis showed higher PDGFRα expression level in HCC with MVI than those without MVI. **(C)** Kaplan-Meier analysis revealed significantly poorer overall survival in HCC with MVI than those without MVI.

Because the above data identified significant correlation between PDGFRα expression and MVI, we further investigated different PDGFRα expression in HCC with and without MVI. IHC staining identified higher PDGFRα expression in primary HCC with MVI than those without MVI (Figure 3A and B, *p*=0.012). Additionally, Patients with MVI had significantly poorer OS than those without MVI after curative surgery for HCC (Figure 3C, log-rank test, *p*<0.001). These data suggests that PDGFRα might play a critical role in vascular invasion and metastasis of HCC.

### High PDGFRα expression is positively associated with increased microvascular density

**Figure 4:**
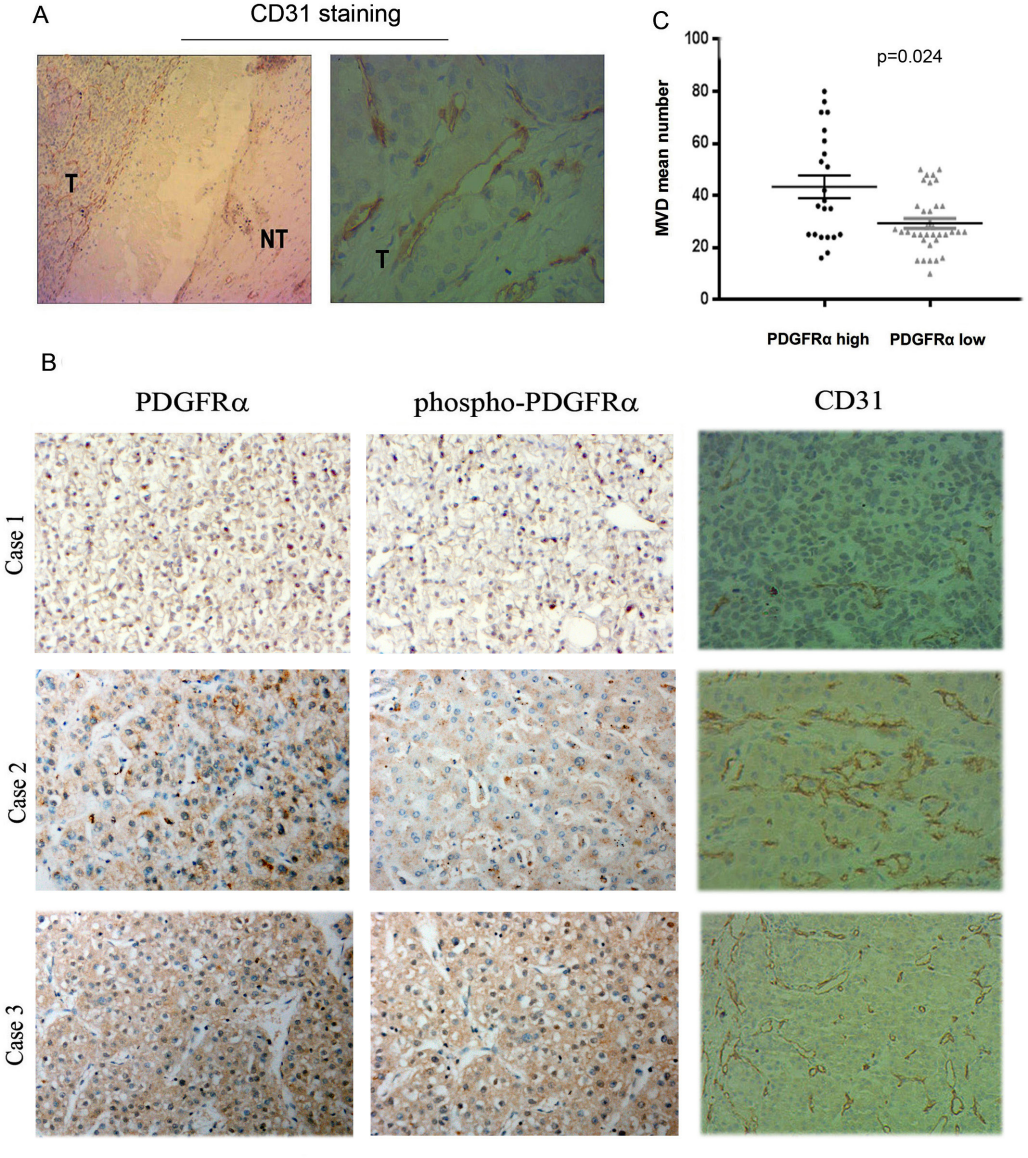
Overexpression of PDGFRα and Tyr^754^-PDGFRα (phosphor-PDGFRα) in human HCC samples correlated with tumor microvessel density (MVD) **(A)**: Representative IHC staining for CD31 showed remarkable positive staining in HCC than the matched normal liver tissues. Original magnification: 4× in the left and 40× in the right panel. **(B)** Representative IHC staining examined increased MVD in HCC with strong PDGFRα staining than those with weak PDGFRα staining. Original magnification: 40×. **(C)** Quantitative analysis confirmed correlation of PDGFRα expression level with MVD in HCC.

Next, we examined whether PDGFRα expression was correlated with microvascular density (MVD) in 57 HCC tissues. Specific staining of capillary-like vessels by anti-CD31 was observed in all tumor specimens. In contrast, there is only sparse staining of microvessels in nontumor liver tissues (Fig. 4A). The MVD in 22 patients with high PDGFRα expression (43.3±4.4/0.74 mm^2^) was significantly higher than those tumors with low PDGFRα expression (29.2±1.9/0.74 mm^2^) (Fig. 4B and C) (*p*=0.024). This relationship between PDGFRα expression and MVD indicated that PDGFRα might be a critical factor promoting angiogenesis in HCC.

### Stable transfection of PDGFRα in Hep3B cells promotes cell proliferation, migration and invasion

**Figure 5:**
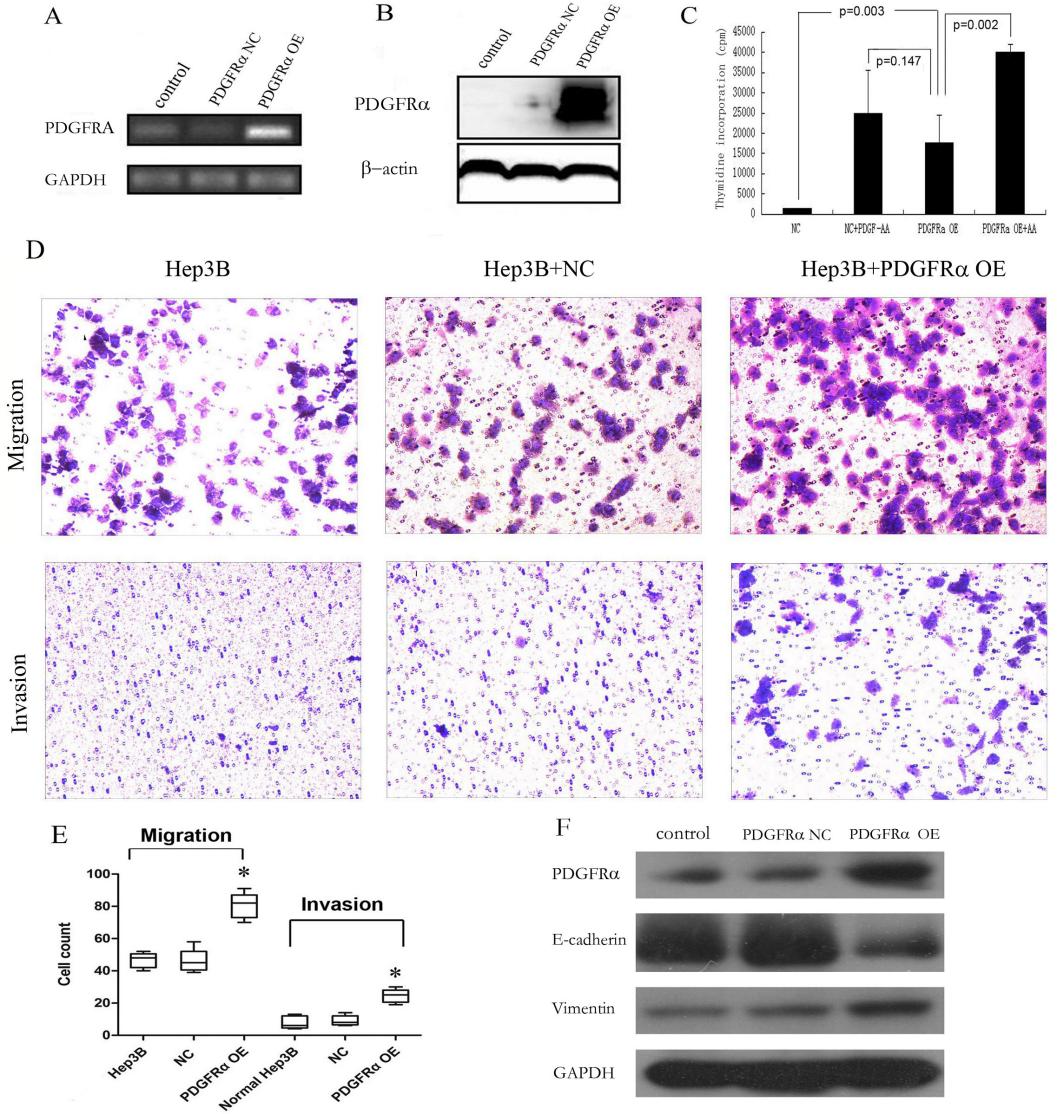
Stable PDGFRα overexpression (OE) in hepatoma cells promoted cell proliferation, migration and invasion (A & B): Semiquantitative PCR **(A)** and immunoblotting **(B)** analysis of PDGFRα expression levels in Hep3B cells stably transfected by negative control (NC) and PDGFRα-lentiviral vector (PDGFRα OE). **(C)**: Cell proliferation was compared by thymidine incorporation assay between cells transfected with PDGFRα NC and PDGFRα OE either with or without addition of PDGF-AA. **(D & E)**: Cell migration and invasion between cells transfected with PDGFRα NC and PDGFRα OE. **(F)** Western blot of E-cadherin and vimentin expression between untreated cells and those transfected with PDGFRα NC or PDGFRα OE. (* *p*<0.05).

*In vitro* experiments were then performed to further address potential impacts of PDGFRα on biological behavior of hepatoma cells. Vectors containing full-length PDGFRα cDNA were constructed successfully (Suppl. Figure 1). Establishment of stable lentiviral infectants with PDGFRα overexpression (OE) was verified at both mRNA and protein levels (Fig. 5A and B). The thymidine incorporation assay was performed to examine the impact of PDGFRα on cell proliferation. Considering that activation of PDGFRα and downstream signaling requires to be stimulated by its ligands, we added PDGF-AA in cell cultures. Significant increases in thymidine uptake by Hep3B cells with PDGFRα OE, PDGF-AA stimulation and both combined (Figure 5C, all *p*<0.05) were examined compared to controls. Conversely, treatment of hepatoma cells SK-Hep1 with PDGFR inhibitor Imatinib could significantly decrease cell viability, proliferation or colony formation (Suppl. Figure 2).

Moreover, significant differences were present in both migration and invasion assays between PDGFRα OE and control group (Fig. 5D and E). Hep3B cells are commonly believed to exert very low metastatic potential, and our results also suggested that normal Hep3B scarcely transverse the Matrigel in invasion assay. But remarkably much more cells with PDGFRα OE passed through transwell membrane either or not coated with Matrigel (Fig. 5D and E).

The underlying mechanism for promoted invasion of Hep3B cells after PDGFRα overexpression was further investigated. We found decreased E-cadherin and increased vimentin expression in cells with overexpressing PDGFRα, indicating that PDGFRα promoted epithelial-mesenchymal transition (EMT) of hepatoma cells (Fig. 5F).

### PDGFRα accelerates tumor growth in vivo

**Figure 6:**
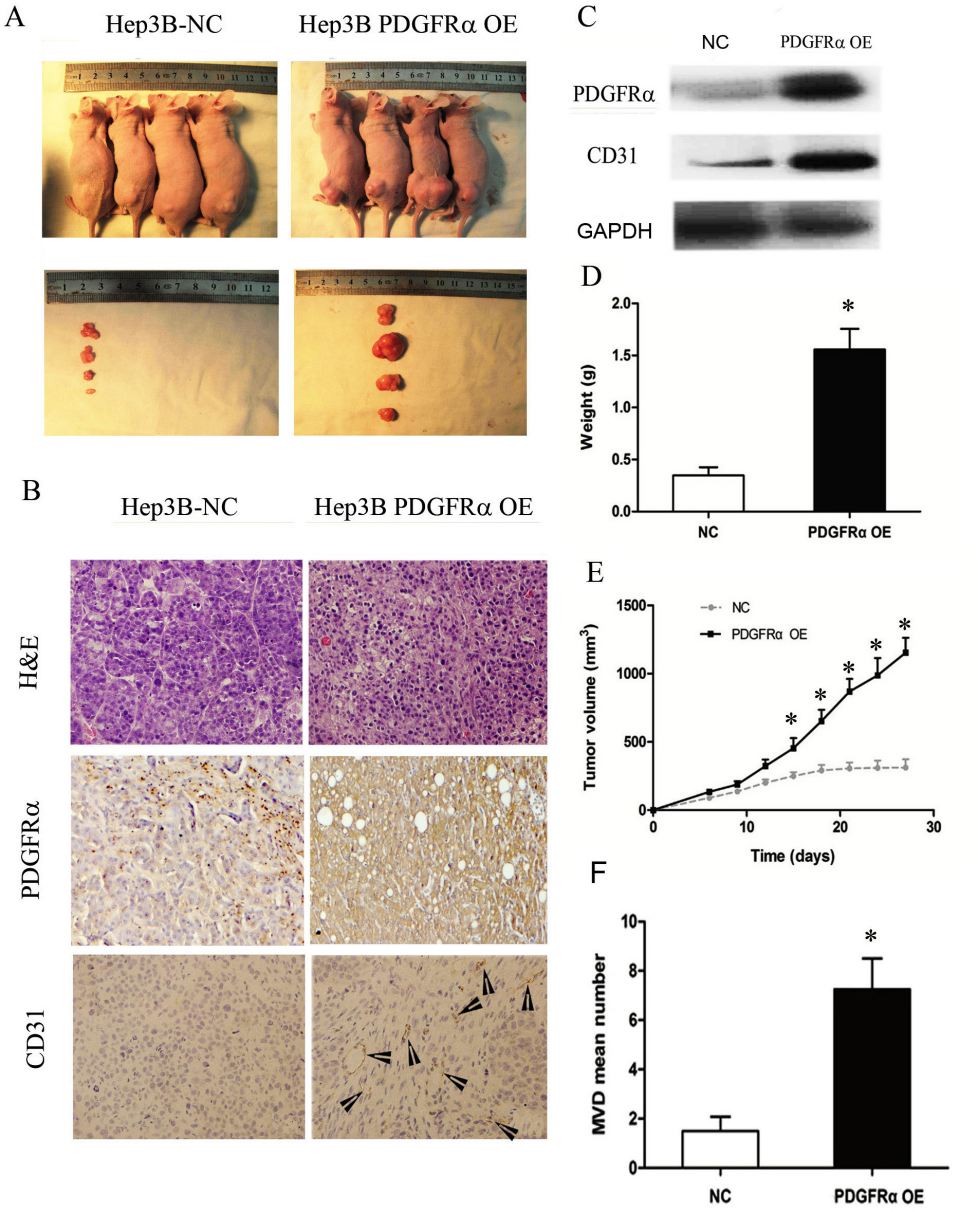
PDGFRα overexpression (OE) promotes tumor growth and progression *in vivo* **(A)**: Tumor nodules after subcutaneously inoculation of Hep3B cells transfected with PDGFRα OE or NC for four weeks (n=6 for each group). **(B)**: H&E staining and immunostaining for PDGFRα and CD31 of tumors with PDGFRα OE or NC transfection. Original magnification: 20×. The arrowheads indicates vessel-like structures positive for CD31 staining. **(C)** Immunoblotting for PDGFRα and CD31 of tumors with PDGFRα OE or NC transfection. **(D & E)**: *In vivo* tumor growth was compared in tumor weight and volume between cells transfected with PDGFRα OE and NC. **(E)** Quantitative comparison of MVD between tumors with PDGFRα OE and NC transfection. (* *p*<0.05).

Subsequently, we undertook experiment by injecting PDGFRα OE and control cells into nude mice subcutaneously to assess the role of PDGFRα in tumorigenesis *in vivo* (Fig. 6A). Tumor xenografts were dissected, the development of tumor lesions was verified by H&E staining. PDGFRα and subsequent CD31 expression was verified by immunohistochemical staining and western blot (Fig. 6B and C). High PDGFRα expression led to substantially increased tumor volume and tumor weight, as well as increased CD31 expression (Fig. 6B, C, D and E). We further measured the MVD by immunostaining for CD31 in each tissue section, and significantly elevated MVD by 6 to 8-fold was noticed in the tumor foci of PDGFRα OE group compared with control group (Fig. 6B and F). These findings further indicated that PDGFRα might promote HCC progression partially through enhancing neovascularization.

## DISCUSSION

PDGFRα has been implicated in physiological development of many organs and multiple diseases [14]. It is widely accepted that PDGFRα is activated in various cancers and serves as a potential therapeutic target [8, 15, 16]. Among patients with gastrointestinal stromal tumors, seven percent cases harbors activating mutations in PDGFRα and targeted therapy inhibiting PDGFRα significantly prolongs survival of these patients [17, 18]. In the present study, we found high expression of PDGFRαprotein in a subset of HCC (~38.6%) compared to adjacent liver tissues, and concomitant enhanced phospho-PDGFRα immunostaining. However, the mRNA level of PDGFRA was decreased in HCC versus normal liver, indicating possible posttranscriptional modification of mRNA to retard its degradation. Although the initiation mechanism of PDGFRα upregulation remains unknown, increase of its ligand, PDGF-A in HCC might indicate presence of autocrine or paracrine loops in hepatocarcinogenesis [8].

Similar to other oncogenes, remarkably high expression of PDGFRα was observed in specific stages during early liver development but a low level is maintained in normal liver through adulthood [8, 11]. By assuming the commonalities across the organogenesis and tumorigenesis, PDGFRα was found increased in HCC, compared to normal liver in some previous studies [8, 9, 19]. And 3G3, a PDGFRα antibody, could inhibit hepatoma cells growth [8], the mechanism and clinical relevance of which, however, remains unknown. The present study identified that high PDGFRα expression was strongly associated with macroscopic vascular invasion, and thus poor OS and DFS of the patients after surgery. In another study, although a strict grading scale had been used, high PDGFRα in HCC was independently correlated with poor prognosis [9]. These clinical data suggests that PDGFRα contributes to malignant progression of HCC and might be a prognostic marker.

The mechanism of PDGFRα promoting HCC development remains obscure. Intriguingly, a recent study demonstrated that PDGFRα staining predominantly located in small blood vessels instead of tumor cells [10]. Indeed, we also observed positive staining in vessel-like structures, suggesting a role of PDGFRα in regulation of endothelial cells. As firstly identified by the present study, both human and mouse HCC tissues with high PDGFRα expression displayed significantly higher MVD than those with low PDGFRα expression, respectively, implying a potential role of PDGFRα in tumor angiogenesis. It is widely established that angiogenesis accounts for a prerequisite for growth and progression of HCC, a typically hypervascular cancer [20]. PDGF possessing similar sequence and structure as vascular endothelial growth factor (VEGF) exhibits pro-angiogenic effects in a wide range of conditions [6]. It has been occasionally found that interaction between PDGF and their receptors involves in angiogenesis of liver cancer but not as intensely studied as VEGF [15]. The transgenic mice conditionally overexpressing PDGF-CC in liver spontaneously developed HCC resembling the successive step of human liver tumorigenesis, and activation of endothelial cells was one of the prominent features [21]. And it was further demonstrated in their study that treatment with imatinib led to attenuation of angiogenesis and tumor growth through inhibiting PDGF signaling [22]. Given that PDGF-CC primarily signals through PDGFRα, it is rational to speculate the potential influence of PDGFRα on hepatocarcinogenesis, especially on neovascularization.

Several pieces of evidence in this study support a close association of PDGFRα expression and HCC progression. First, PDGFRα expression was significantly higher in HCC with MVI than that in HCC without MVI. Second, HCC with high PDGFRα expression displayed higher MVD than those with low PDGFRα expression. Third, overexpression of PDGFRα in hepatoma cells promotes cell proliferation, migration,invasion and EMT *in vitro* and tumor growth *in vivo*. It was previously suggested that PDGFRα overexpression on microvessels was associated with high metastatic potential in HCC cases [7]. Consistently, PDGFRα upregulation was observed on endothelial cells from highly metastatic tumor xenografts and appeared to have a predictive value for HCC recurrence [7]. This finding was corroborated in another clinical study that PDGFRα overexpression in the endothelial cells of HCC tissues was associated with microvascular invasion and high recurrence rate [10]. Interestingly, our *in vitro* study showed that PDGFRα overexpression in hepatoma cells instead of endothelial cells also resulted in increased metastatic propensity. Further examination of its mechanism revealed that induction of EMT upon PDGFRα upregulation accounted for the enhanced tumor cells invasion, and thus compromised prognosis of HCC patients, as suggested by a recent study that EMT is involved in HCC recurrence after surgery [23]. PDGF-D, a ligand of PDGFR, has been found inducing chemoresistance of HCC via promoting EMT phenotype of cancer cells [24].

In conclusion, this study demonstrates that overexpression of PDGFRα in HCC is an indicator of aggressive behavior of tumors and poor clinical outcomes of patients. Therefore, PDGFRα expression may be a candidate biomarker and therapeutic target of HCC.

## MATERIALS AND METHODS

### HCC specimens, cell lines and chemical reagents

30 and 27 pairs of surgically resected HCC and adjacent liver tissues were collected from the 1st and the 2nd Affiliated Hospital of Medical College, Xi'an Jiaotong University, respectively. All 57 pairs of samples were formalin-fixed and paraffin embedded for subsequent immunohistochemical examinations. Another 14 pairs of freshly resected specimens of HCC and adjacent normal liver tissues (at least 3cm away from the tumor edges) were collected from the 1st Affiliated Hospital above. The tumor and adjacent normal liver tissues were cut into small pieces and frozen in liquid nitrogen, then store in −80°C refrigerator for further protein analysis. The research was approved by the Ethics Committees of Xi'an Jiaotong University and informed consents were obtained from all enrolled patients.

Human liver cell line HL-7702, HCC cell lines Huh7, HepG2, Hep3B, Snu387, Snu449 and SK-Hep1 were purchased from Shanghai Institute for Biological Science, Chinese Academy of Sciences (Shanghai, China). Cells were cultured in high glucose Dulbecco's modified Eagle's medium (DMEM) or Roswell Park Memorial Institute (RPMI) media 1640 (Hyclone, USA) containing 10% fetal bovine serum (Hyclone, USA) and 1% penicillin/streptomycin (Hyclone, USA) and were maintained under the circumstance of 37°Cand 5% CO_2_ in a humidified chamber.

Human recombinant PDGF-AA was from PeproTech (USA). The white compound was dissolved in ddH_2_O at 20ug/ml and was stored at −80°C. A concentration of 50ng/ml diluted in culture medium was used in the experiment, unless otherwise stated [25].

### Establishment of construct, stable infection and clone selection

Full-length of wild-type PDGFRA cDNA fragment was synthesized by reverse transcriptase-PCR and amplified (Suppl. Figure 1A). GV-166 vector containing a puromycin selection marker was obtained commercially from Gene Chem (Shanghai, China) (Suppl. Figure 1B). Considering the long sequence of full-length PDGFRA may affect the production of lentiviral particles, the GFP was not added to make the length of vector. The fragment described above was then subcloned into GV166 vector at BamHI/AgeI site and positive product was verified by PCR (Suppl. Figure 1C). The primers for PDGFRA (forward: 5′-AATTCCGTGGTGTTGTCG-3′; reverse: 5′-AAGGTCCGCTGGATTGAG-3′). Then the DNA sequencing confirms the generation of positive clone. In addition, 293T cells were transfected by the construct and western blot show the successful expression of infusion gene (Suppl. Figure 1D and E). The lentiviruses were produced by co-transfecting vector containing gene encoding PDGFRα and packaging plasmids into 293T cells using Lipofectamin 2000. The supernants were collected 48 hours after transfection and filtered via a 0.45-um filter to acquire the lentiviral particles. For negative controls, empty vector construct containing GFP was adopted. Hep3B cells were infected by extracted lentiviruses at multiplicity of infection (MOI) of 50 and then selected by 2ug/ml puromycin for 4 weeks. The parallel experiment using control lentiviral vector particle was undertaken and the observation of fluorescence within cells indicated successful infection (Suppl. Figure 1F). Expression of exogenous PDGFRα in Hep3B cells was verified by RT-PCR and western blot.

### Immunohistochemistry and assessment of microvessel density

The prepared 5 um thick paraffin serial sections were deparaffinized by xylene, rehydrated and blocked by 3% hydrogen peroxide. The following primary antibodies were used to incubate sections overnight at room temperature: PDGFRα (1:50, Santa Cruz), Tyr^754^-PDGFRα (phosphor-PDGFRα, 1:50, Santa Cruz), CD31 (Abcam, 1:100). The sections were stained with biotin-conjugated secondary antibodies and tertiary antibodies conjugated to streptavidin peroxidase. Thereafter, 3,3′-diaminobenzidine staining was done and sections were subsequently counterstained with hematoxylin. Finally, all slides were viewed under the microscope. The percentage of positive cells was divided into four grades (percentage scores): 10% (0), 10-25% (1), 25-50% (2), 50-75% (3). The intensity of staining was divided into four grades (intensity scores): no staining (0), weak staining (1), moderate staining (2) and strong staining (3). PDGFRα staining positivity was determined by the formula: overall score = percentage score × intensity score. The overall score ≤3 was defined as negative, and ≥4 was defined as positive.

Microvessel density (MVD) of tissue sections were assessed by two independent observers according to methods described before [26]. Microvessels were defined as brown CD31-immunostained tube-like structure or cell cluster. The sections were screened at 40× field to select five areas with most intense angiogenesis. At high power field (200×), the definite number of microvessels per 0.74 mm^2^ were counted. The mean microvessel number of five selected areas was considered as MVD.

Additional methods are provided in the Supporting Information.

### Statistical analysis

All in vitro experiements were performed at least 3 times and in triplicate for each individual experiment. All quantitative data were presented as mean ± standard deviation (S.D.) and were analyzed statistically using one-way ANOVA or 2-sample t test. All categorical variables were expressed as percentage, and were analyzed statistically using Pearson's Chi-square test or Fisher's exact test. Survival curves were plotted using Kaplan-Meier method, and the differences of survival rate were evaluated by the log-rank test. p<0.05 was considered statistically significant. All calculations and analysis were performed using the SPSS statistic software 13.0 (SPSS, Chicago, IL).

## SUPPLEMENTARY METHODS AND FIGURES


