# Supplementary material for: Overexpression of platelet-derived growth factor receptor alpha promotes tumor progression and indicates poor prognosis in hepatocellular carcinoma
Source: Oncotarget. 2014 Oct 17;5(21):10307–17. doi: 10.18632/oncotarget.2537 (PMC4279374; doi:10.18632/oncotarget.2537)
Supplement: Supplementary file 1 [file oncotarget-05-10307-s001.pdf]

# SUPPLEMENTARY METHODS AND FIGURES

## Immunoblotting

Fresh HCC specimens and hepatoma cells were washed with ice-cold phosphate-buffered saline (PBS) and lysed using cell lysis buffer with protease inhibitor (Roche). The protein concentrations were determined using bicinchoninic acid method (Pierce, Rockford, IL). Equivalent amounts of protein were separated by 10% sodium dodecyl sulfate-polyacrylamide gel electrophoresis and transferred into polyvinylidene difluoride membrane (Millipore, Billerica, MA). Membranes were blocked in tris-buffered saline/0.1% Tween 20 with 5% nonfat milk. Afterwards, the primary antibodies for PDGFRα (Santa Cruz; Sc-338, 1:200), E-cadherin (Cell Signaling; 3195, 1:1000), vimentin (Cell Signaling; 5741, 1:1000) and GAPDH (Santa Cruz; Sc-365602, 1:200) were applied to incubate membranes overnight at 4°C. The membranes were washed 10 minutes for 3 times and then incubated with secondary antibodies (1:5000, Pioneering Biotechnology, China) for 1 hour at room temperature. After washing, the blots were visualized using Immobilon ECL Chemiluminescent (Millipore, Billerica, MA).

## Quantitative reverse transcription-PCR

HCC and paired nontumor tissues were lysed in TRIzol Reagents (Invitrogen) to obtain total RNA. Then, extracted RNA was reverse transcribed using Prime Script reagent RT Kit (Takara Biotechnology, Dalian, China). Real-time qPCR was underwent on the ABI 7900 Prism HT (Applied Biosystems, Shanghai, China), and delta CT method was adopted to calculate the fold change in gene expression levels between groups. The primers were designed by Takara as follows: PDGFRA(forward: GGTCTTATGGCATTCTGCTCTGG; reverse: CTTCACTGGTAGCGTGGTCAGG), PDGFA (forward: ACTAAGCATGTGCCCGAGAA; reverse: GTAAATGACCGTCCTGGTCTTG), PDGFC (forward: CTGGCGGTGGTGAAAGAGA; reverse: AGCAGGAGAAGCCCGAAGA), β-actin (forward: TGGCACCCAGCACAATGAA; reverse: CTAAGTCATAGTCCGCCTAGAAGCA).

## MTT, thymidine incorporation assay

MTT assays were used to assess the viability of Hep3B cells with overexpression of PDGFRα. The amount of 2000 cells per well were seeded in 96-well plates and the cells were stimulated with or without PDGF-AA. Afterwards, media were removed and MTT were added into each well for additional 4 hours. Media were removed and formazan crystals were dissolved with 200ul dimethylsulfoxide (DMSO). Optical density (OD) was measured at 490 nm on a Multifunction Microplate Reader. Each experiment consisted of five replications and at least three independent assays were carried out.

For the thymidine incorporation assay, thymidine [$^3$H] (2.5 μCi/ml) was added to medium in the hepatoma cells with control or PDGFRα OE. Cells were harvested after 24 hrs and assayed for thymidineincorporation as described [11].

## Ectopic xenograft model

Male BALB/c nude mice (4 weeks old) were obtained commercially from the Shanghai Experimental Animal Center and were bred in laminar-flow chambers under specific pathogen-free conditions in the Laboratory Animal Center of Xi'an Jiaotong University. The animal research was in accordance with University Institutional care and Use Committee. Our preliminary study found that normal Hep3B cells have low tumorigenic potential in ectopic xenograft model in a cell density of $5\times10^7$/ml. To investigate whether cells overexpressing PDGFRα gain increased tumorigenic capacity over negative control, a total of $1\times10^7$ cells in 200ul serum-free culture medium were injected subcutaneously into the left region of each mouse. Twenty animals were divided evenly into two groups receiving either PDGFRα overexpressing (PDGFRα OE) cells or control cells. The mice were observed every 3 days and their weight were recorded. Meanwhile, the tumor size was calculated using equations of $(a\times b^2)/2$, wherein a and b represent the longest and shortest diameter, respectively. Mice were sacrificed by cervical dislocation 4 weeks later, and tumor xenografts were dissected and weighted. Each tumor sample were fixed in 4% paraformaldehyde and paraffin embedded.

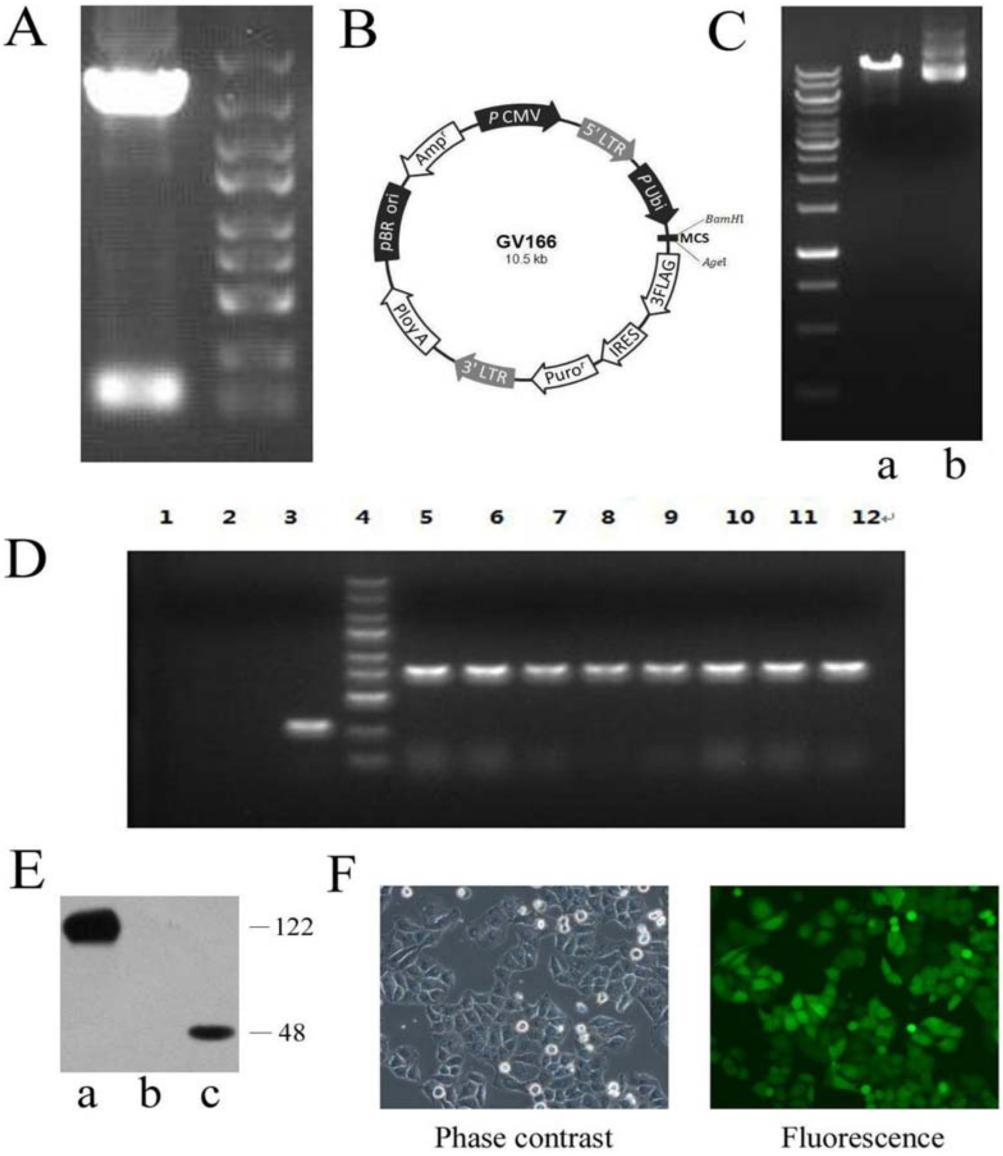


**Supplementary Figure S1: (A): Full-length of wild-type PDGFRA cDNA fragment was synthesized.** **(B)**: Scheme of GV-166 vector. **(C)**: The wild-type PDGFRA cDNA was subcloned into GV166 vector at BamHI/AgeI site (a) and positive product (b)was verified; **(D)**: Construction of recombinant plasmid. 1. negative control (ddH2O); 2. negative control (empty vector); 3. positive control (GAPDH); 4. marker; 5-12. 1-8 transformants of PDGFRA. **(E)**: Immunoblotting showed expression of infusion gene (a), negative control (b) and SURVIVIN-3FLAG-GFP as positive control (c). **(F)**: Observation of fluorescence within cells infected by control lentivirus.

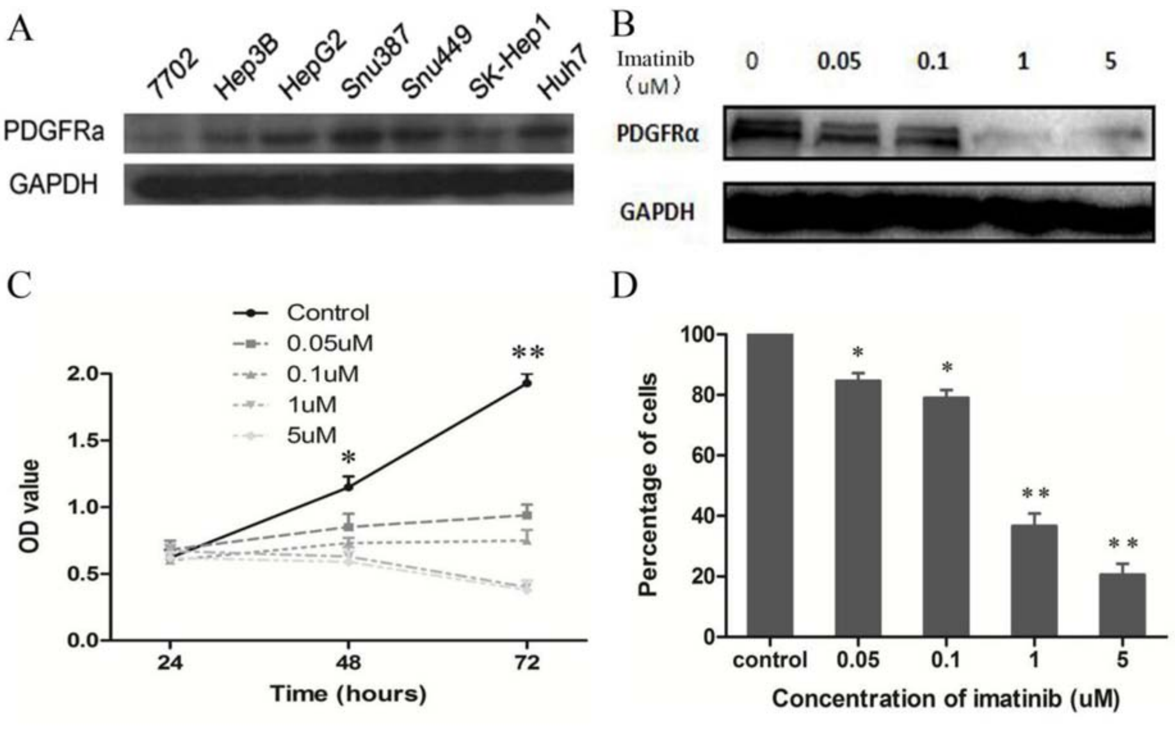


**Supplementary figure S2: Effects of imatinib on inhibition of PDGFRα expression and hepatoma cell proliferation (A)**: Expression of PDGFRα protein in normal liver cell line HL-7702 and six hepatoma cell lines. **(B)**: Expression of PDGFRα in SK-Hep1 after treatment with different dose of imatinib. **(C)**: MTT assays displaying the number of viable cells in control cell line and hepatoma cells treated with different dose of imatinib. **(D)**: Colony formation of control and cells treated with different dose of imatinib. (* p<0.05, ** p<0.01).
